# Improving Inclusion of Ethnically Diverse and Socioeconomically Disadvantaged Populations in Pain Research: A Comprehensive Review and Evidence-Based Recommendations

**DOI:** 10.3390/ijerph23081091

**Published:** 2026-08-21

**Authors:** Kevin Pacheco-Barrios, Allison Kim, Carla Pastora-Sesin, Joao Mariano, Robin Heemels, Paulo S. de Melo, Erick Barrientos-Ventura, Lucas Camargo, Niels Pacheco-Barrios, Jaime Pacheco-Neyra, Silvia Di-Bonaventura, Raúl Ferrer-Peña, Alba Navarro-Flores, Equity in Pain Research Working Group (EPR-WG)

**Affiliations:** 1 Neuromodulation Center and Center for Clinical Research Learning, Spaulding Rehabilitation Hospital and Massachusetts General Hospital, Harvard Medical School, Boston, MA 02138, USA; 2 Unidad de Investigación para la Generación y Síntesis de Evidencias en Salud, Vicerrectorado de Investigación, Universidad San Ignacio de Loyola, Lima 15023, Peru; 3 Admission AG, Irvine, CA 92618, USA; 4 Department of Neurosurgery, Mass General Brigham, Harvard Medical School, Boston, MA 02215, USA; 5 Research Unit, Our Lady of Chapi Pharmacy, Cajamarca 06001, Peru; 6 Department of Physical Therapy, Occupational Therapy, Rehabilitation and Physical Medicine, Rey Juan Carlos University, 28922 Alcorcon, Spain; 7 Research Unit, Bei App, 28023 Madrid, Spain; 8 Clinical and Teaching Research Group on Rehabilitation Sciences (INDOCLIN), CSEU La Salle, Universidad Autónoma de Madrid, 28023 Madrid, Spain; 9 Centro de Salud Entrevías, Gerencia Asistencial de Atención Primaria de la Comunidad de Madrid, 28023 Madrid, Spain; 10 Institute of Psychiatric Phenomics and Genomics (IPPG), LMU University Hospital, LMU Medizin, Ludwig-Maximilians-Universität München, 80336 Munich, Germany; 11 International Max Planck Research School for Translational Psychiatry (IMPRS-TP), 80804 Munich, Germany

**Keywords:** pain, ethnically diverse populations, socioeconomically disadvantaged populations, recruitment, retention, clinical trials

## Abstract

**Highlights:**

**Public health relevance—How does this work relate to a public health issue?**

- Chronic pain is highly prevalent among ethnically diverse and socioeconomically disadvantaged populations, yet these groups remain systematically underrepresented in pain research, limiting external validity and equity in evidence generation.
- This review addresses a critical public health gap by examining recruitment and retention strategies that shape who is represented in pain research and, ultimately, who benefits from evidence-based pain care.

**Public health significance—Why is this work of significance to public health?**

- The study demonstrates that while enrollment of disadvantaged populations in pain research is low, retention is consistently high once participants are enrolled, challenging assumptions about feasibility and adherence.
- By synthesizing quantitative and qualitative evidence, this work provides the first evidence-based comparison of recruitment and retention strategies specifically effective for marginalized pain populations.

**Public health implications—What are the key implications or messages for practitioners, policy makers and/or researchers in public health?**

- Community-engaged recruitment approaches, clinician referrals, culturally and linguistically tailored materials, and flexible hybrid study designs can substantially improve equitable participation in pain research.
- Standardized reporting of recruitment and retention metrics by strategy should be prioritized in research policy and funding requirements to strengthen equity-focused trial design and implementation.

**Abstract:**

Limited inclusion of ethnically diverse and socioeconomically disadvantaged populations in pain research undermines external validity, generalizability, and equity. This comprehensive review synthesized evidence on effective strategies to recruit and retain these populations in pain studies. We searched Medline, Web of Science, Embase, Scopus, and CENTRAL (12 April 2025) and included studies in which ethnically diverse and socioeconomically disadvantaged participants comprised ≥75% of the sample. Quantitative data were pooled using random-effects meta-analyses of proportions, with prespecified subgroup analyses, and qualitative findings were integrated through thematic synthesis. Certainty of evidence was evaluated using GRADE. Eighteen studies (n = 4611; 11 experimental, 5 observational, 2 qualitative), primarily from the United States and involving chronic pain, met inclusion criteria. Overall enrollment among ethnically diverse and socioeconomically disadvantaged groups was 37% (95% CI 18–58%), with significantly higher enrollment in observational studies (84%, 95% CI 78–90%) than in experimental trials (22%, 95% CI 10–36%; *p* < 0.001). Overall retention was 77% (95% CI 64–88%) and did not differ significantly by study design. Statewide disease-clinic networks, purposive community-leader engagement, and snowball sampling produced the highest enrollment, whereas medical-record screening yielded the lowest enrollment but the highest retention. Compensation and reminder strategies were similarly effective for retention. Thematic synthesis highlighted trust, culturally and linguistically tailored communication, hybrid and flexible visit schedules, transportation assistance, and clinician engagement as key facilitators. GRADE certainty was low for enrollment and moderate for retention. Community-engaged recruitment strategies, clinician referrals, culturally tailored materials, hybrid procedures, and modest incentives can substantially improve participation of ethnically diverse and socioeconomically disadvantaged populations in pain research. Standardized CONSORT-style reporting of recruitment/retention flowcharts according to strategy and ethnicity/socioeconomic status is essential to refine evidence-based strategies in the future.

## 1. Introduction

Pain is defined as “an unpleasant sensory and emotional experience associated with, or resembling that associated with, actual or potential tissue damage” [1]. This experience can be mainly divided into acute or chronic, the latter being the persistence of pain for more than three months [2]. While the acute state of pain is unpleasant by itself and correlated with negative emotions [3,4,5,6], chronic pain is considered an independent medical condition, estimated to be present in up to 40% percent of the USA population [7]. This condition is a widespread public issue, presenting in a diverse range of the population. However, there is a critical gap between the proportion of ethnically diverse and socioeconomically disadvantaged populations in the general population and their representation in clinical research. For example, a 2010 survey estimated that 7.6% of the population included in NIH studies were part of the minority group of Hispanics, while the percentage of that minority in the general population was estimated to be above 16% in that same period [8]. This difference can create heterogeneity of the estimated treatment effects, affecting the external validity of the clinical pain studies.

The genesis of this underrepresentation might be due to a mixture of factors, such as limited access to healthcare, limited work offer, higher exposure to adverse and informal work environments, and the wage difference between ethnicities. It is estimated that black workers earn approximately 81 cents and Hispanic or Latino workers approximately 77 cents for every dollar earned by White workers. [9]. Thus, disadvantaged populations need to work more hours to generate the same income, being less available for participation in clinical research. On top of that, in 2018 more than 25 million people in the USA spoke English “less than very well” [10], while most of the studies have English-speaking staff and English written documents. Therefore, language and cultural barriers must also play an important role in the underrepresentation of these populations in research. Moreover, well-established and clear communication is essential to build trustful and reliable relationships between research staff and recruited subjects.

Pain populations face other challenges inherent to the unpredictable course of their health conditions [11]. The oscillatory nature of their symptoms, disease severity, and comorbidities, such as depression, fatigue, and anxiety [12], might put them at increased risk of not enrolling or not adhering to the protocol—either due to the emergence or worsening of symptoms or to the transient amelioration of those symptoms. The lack of representation of ethnic and socioeconomic diverse populations in pain studies not only affects the validity and implementation of new pain treatments, but also importantly affects our mechanistic understanding of pain chronification and analgesia, since recent evidence suggests that genetic ancestry and socioeconomic determinants (e.g., discrimination) could play an important role in chronic pain risk and maintenance [13,14]. Hence, multidisciplinary and multisectoral efforts are needed to optimize and promote participation of disadvantaged populations in pain-related research.

Even though this is a self-evident scenario, the literature is insufficient and not yet synthesized on the best approaches to target and maintain the adherence of these populations in pain-related clinical research. Therefore, we aimed to comprehensively assess the evidence on recruitment and retention of ethnically diverse and socioeconomically disadvantaged populations in pain-related clinical research, to identify, describe, and analyze the recruitment and retention methods, barriers and facilitators, and provide evidence-based recommendations on how to improve their representation.

## 2. Methods

We followed the recommendations from the Cochrane handbook [15] and the PRISMA guidelines for systematic reviews and meta-analysis (Supplementary Material File S2) [16]. The extraction and evaluation were conducted by two researchers and verified by a third reviewer.

### 2.1. Literature Search and Study Selection

We searched Medline, Web of Science, Embase, Scopus and Cochrane Central (12 April 2025), using combinations of three groups of terms and their synonyms: (1) “Underrepresented” or “Underserved” or “Disadvantaged” or “Ethnic*”; (2) “Recruitment barriers” or “Recruitment strategies”; and (3) “Chronic pain”, “Experimental pain”, “Pain”, and specific pain disorders. The full research strategy is shown in Supplementary Material File S3. Duplicates were eliminated before selection using EndNote X9. Three authors screened the record initially by titles and abstracts and then by full text. Discrepancies between them were resolved by a third author.

### 2.2. Eligibility Criteria

After the search, we included in our review studies of any design, language, or pain condition (acute and chronic), and that gave information about the recruitment process of ethnically diverse and socioeconomically disadvantaged populations (the participant characteristics, recruitment and retention strategies) and recruitment metrics (enrollment or retention rates at least). Ethnic diversity was operationalized as ethnicities other than white. Socioeconomic disadvantage was operationalized according to each primary study’s criteria, most commonly using income-based indicators (e.g., household income below federal poverty level), education level (e.g., less than high school completion), insurance status or safety-net care (e.g., Medicaid-only coverage, recruitment from public clinics), or area-level/composite indices (e.g., high-poverty neighborhoods, Area Deprivation Index) [17,18]. Several studies combined multiple indicators (e.g., low income plus public insurance). Where studies reported recruitment or retention disaggregated by specific ethnic groups (e.g., Black vs. Hispanic participants) or SES markers, we extracted these as subgroup data for descriptive synthesis, though formal meta-analysis by ethnicity or SES was precluded by heterogeneous definitions and sparse reporting.

We included studies reporting mixed samples with at least 75% of disadvantaged populations. Consistent with prior equity-focused systematic reviews, we required that at least 75% of participants be ethnically diverse and/or low-income. Previous reviews examining interventions in marginalized populations have commonly applied majority thresholds (≥75%) to ensure that findings are truly representative of the target groups and to minimize misclassification of mixed samples [19,20,21]. This threshold was selected to ensure that recruitment and retention estimates primarily reflected the experiences and participation patterns of the populations of interest, minimizing the influence of predominantly represented populations while acknowledging that this choice may reduce the generalizability of the findings to more heterogeneous study populations. Opinion papers, editorials, letters for the editors, and studies reporting only recruitment information about the overall population composed of less than 75% of disadvantaged populations were excluded.

Studies involving both acute and chronic pain populations were included because the objective of this review was to evaluate recruitment and retention processes rather than treatment effectiveness. Although recruitment dynamics may vary according to pain duration and clinical context, many structural, socioeconomic, and logistical barriers to research participation are shared across pain conditions.

**Figure 1:**
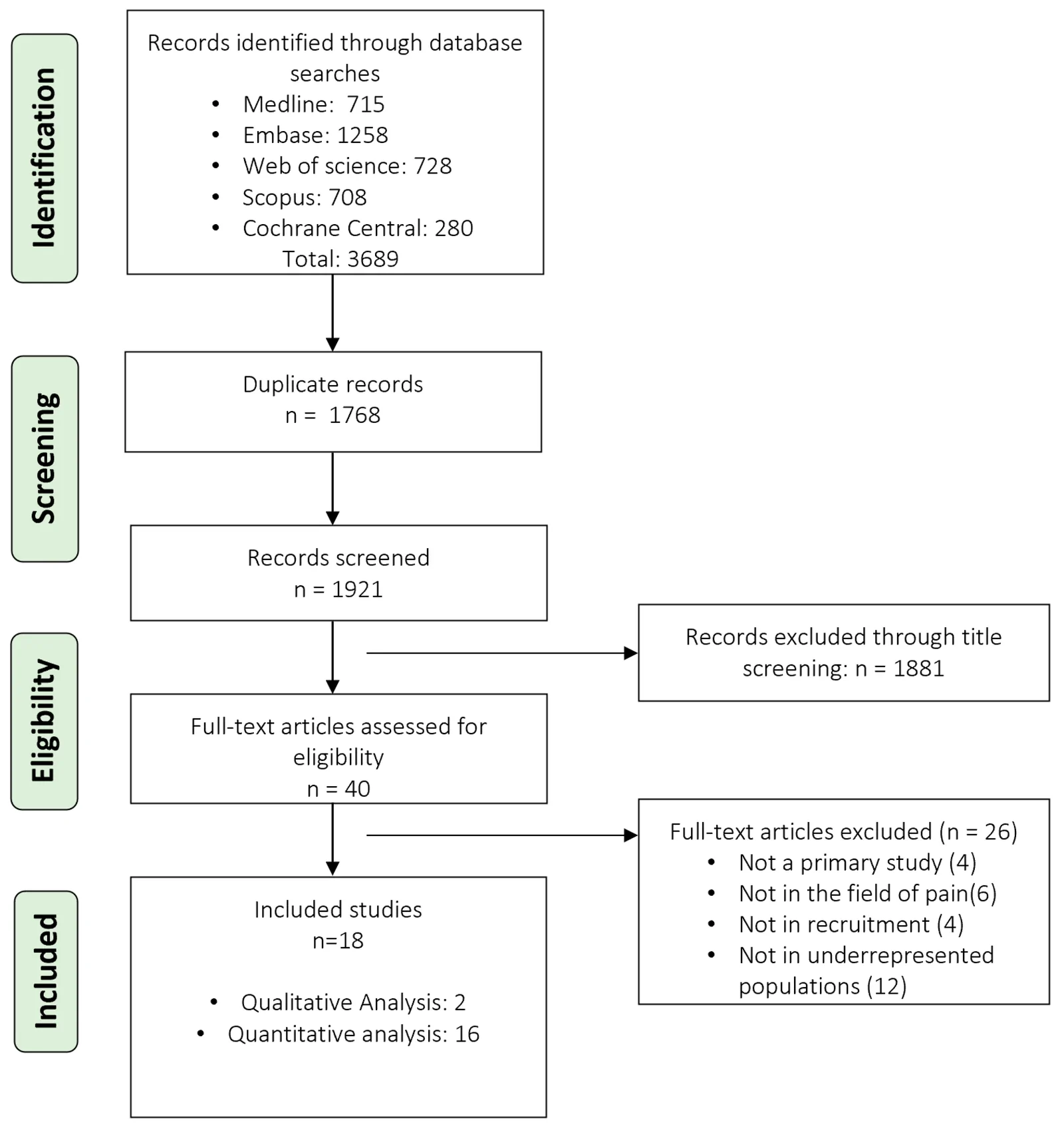
Flowchart of the included studies.

A total of 40 articles were identified during the title and abstract screening and the full text assessed. We included 18 studies [22,23,24,25,26,27,28,29,30,31,32,33,34,35,36] that reported recruitment strategies and that had the majority of the sample recruited as disadvantaged populations (≥75%) (Figure 1). The excluded studies and reasons are shown in Supplementary Material Table S1.

### 2.3. Data Extraction

The extraction of the data was conducted by two researchers. We used a structured table, including information about the year of publication, the number of patients evaluated, study population characteristics, type of enrollment and retention strategies, enrollment and retention metrics, and barriers and facilitators in the recruitment process. The data were tabulated, coded in excel, and then imported into RStudio version 1.4.1106 [37] for analysis.

### 2.4. Risk of Bias Assessment

The included studies risk of bias evaluation was performed using the revised Cochrane risk-of-bias tool, RoB 2.0 (for RCTs) [38] and Newcastle–Ottawa Quality Assessment Scale (NOS) (non-RCTs) [39]. The RoB 2.0 tool evaluates five domains: (a) randomization process; (b) intervention process; (c) missing outcome data; (d) measurement of the outcome; (e) selection of the reported result. The NOS consists of 8 items that evaluate the selection, comparability and outcome or exposure. One star is allocated when a feature of quality is present, up to a maximum of nine (the comparability domain can score up to two stars) [39]. Because the objective of this review was to evaluate recruitment and retention rather than intervention efficacy, the RoB 2.0 and Newcastle–Ottawa Scale (NOS) were adapted accordingly. Domains specifically related to treatment-effect estimation (e.g., blinding and deviations from intended interventions) were considered not applicable when they had no plausible influence on recruitment or retention outcomes, whereas domains relevant to participant selection, outcome measurement, missing data, and selective reporting were retained. For qualitative studies, we used the Critical Appraisal Skills Programme (CASP), which is a recommended tool when conducting health-related qualitative syntheses [40,41]. We classified studies as being of high, medium or low quality if ≥8, 5–7, or ≤4 of the questions in the CASP tool could be answered positively (‘yes’ or ‘somewhat’), as performed in previous research [42]. The assessment was performed by 2 groups of 2 independent investigators (AM, JP, KVA, and IRS), and discrepancies between them were addressed by a fifth reviewer (KP).

### 2.5. Synthesis

A mixed-methods approach was selected because recruitment and retention are influenced not only by measurable outcomes, such as enrollment and retention rates, but also by context-dependent factors that shape participants’ decisions to engage in research. While quantitative analyses identify the effectiveness of recruitment and retention strategies, qualitative evidence provides a deeper understanding of participants’ experiences and uncovers barriers and facilitators that are difficult to capture using standardized quantitative measures alone. Integrating both forms of evidence therefore enabled a more comprehensive interpretation of trial participation.

Accordingly, the evidence was synthesized separately according to study design (qualitative vs. quantitative) and subsequently integrated across methodologies to provide an integrated interpretation of the findings [43].

*Qualitative synthesis:* We used a thematic synthesis [44] of the data reported in the qualitative study. First, we conducted an open-ended coding on each text-unit such as sentence or paragraphs of including studies, focusing on coding “raw” participant data such as quotes, and also coded analyses and conclusions stated by the authors [44]. We performed an iterative process, to code broad and more defined themes on enrollment, retention experiences during pain studies, and any facilitators or barriers identified.

*Quantitative synthesis:* We calculated the exploratory pooled overall enrollment (enrolled subjects/screened subjects) and retention rates (subjects that completed/enrolled subjects) of disadvantaged populations from the studies. Furthermore, we calculated the enrollment and retention rate per strategy, and the retention rate per recruitment strategy. Both proportions were calculated with their corresponding 95% CI, using a binomial model (metaprop command in the meta package in RStudio). We applied the Freeman–Tukey Double Arcsine transformation to stabilize the proportion variance before analyses [45]. These meta-analyses were performed only with exploratory purposes for hypothesis generation, and by a pre-specified random-effects model according to the DerSimonian and Laird method [46] (since high heterogeneity between studies was expected) and the CI calculations were based on the exact method [47]. We assessed the presence of between-study heterogeneity using I^2^ statistics [48]. To evaluate the sources of heterogeneity among included studies, we carried out subgroup analysis by study design (clinical trial and observational studies) and studies with mixed population (75–99% vs. 100% of disadvantaged participants included in the studies).

### 2.6. Certainty of the Evidence Assessment

We assessed the certain of evidence similarly to our previously published articles [49,50,51,52] but adapted for a proportion meta-analysis using three of the five domains described in the GRADE handbook: imprecision (range of the CI), indirectness (generalizability of results), and inconsistency (between-study heterogeneity) [53]. Sample size was not used as part of imprecision since the number of participants included per se was not relevant for the analysis but the proportion recruited. Publication bias was not applicable, since no study is designed and published for the purpose of recruitment, Egger’s tests/funnel plots are unreliable for single proportions (symmetrical distributions, no effect directionality), and few studies were included in the meta-analyses (<10). Comprehensive 5-database searches minimized selection bias. This study has limitations, since the risk of bias of the primary studies assessed deviations that occur only after recruitment is completed. Domain-specific risk-of-bias (ROBINS-I for non-randomized studies) was assessed separately from GRADE domains. Indirectness was rated low because recruitment/retention facilitators like trust-building, cultural tailoring, and access supports represent generalizable equity mechanisms applicable beyond U.S./chronic pain contexts. We scored the evidence as high, moderate, low, or very low certainty [54]. We manually adapted a summary of findings table (SoF) from the GRADE online tool (http://gradepro.org) to display the results.

## 3. Results

### 3.1. Description of the Included Studies

Of the 18 included studies, 11 were experimental, 5 observational, and 2 qualitative. Six studies reported 100% of the sample as disadvantaged populations. Sixteen (89%) of the included studies enrolled chronic pain patients. Almost all of them were conducted in the USA and one was conducted in Norway. The recruitment strategies identified were medical records, referral, media, mailing, snowball, purposive, community sites, community events, email, and previous studies. Identified retention strategies included compensation (money and gift card) and visit reminders. A detailed description of the included studies is presented in Table 1.

### 3.2. Quantitative Analysis

The data were reported in a highly heterogeneous manner, so separate estimates for each disadvantaged subgroup could not be extracted and therefore pooled independently, so average estimates per study were used.

#### 3.2.1. Enrollment

**Figure 2:**
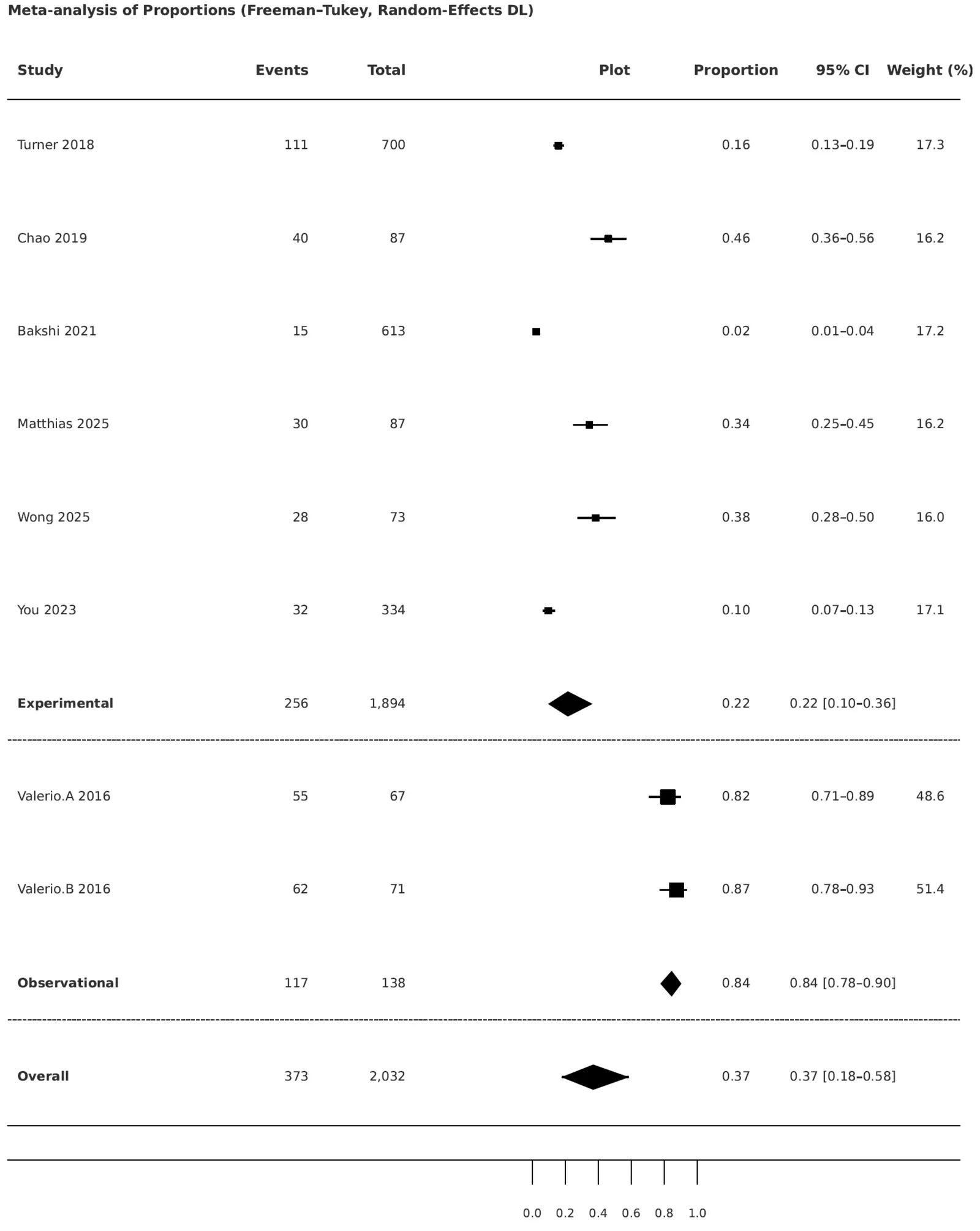
Forest plot of enrollment rate per study and per study design [23,24,31,34,35,36,59].

We analyzed the proportion of the screened and enrolled disadvantaged (enrollment rate) subjects in the included studies with more than 75% of the sample as a disadvantaged population. The general enrollment rate was 37% (95% CI 18–58%). The enrollment rates of the experimental (22, 95% CI 10–36%) and the observational (84%, 95% CI 78–90%) studies were statistically different (*p* < 0.001) (Figure 2).

Furthermore, we performed a sensitivity analysis between the studies that had 100% and 75% of the sample based on a disadvantaged population. The enrollment rate in the studies that had 100% of the sample based on a disadvantaged population was 10.09% (95% CI 0.65–28.43%) and in those with 75% the enrollment rate was 54.19% (95% CI 29.49–77.85%), *p* = 0.004 (Supplementary Material Figure S1).

Additionally, we analyzed the enrollment rate by recruitment method. State-wide specific disease clinic network, purposive community leader strategy, and snowball recruitment have demonstrated the most efficiency with higher enrollment rates (89, 87.3 and 82.2, respectively), while using medical records was the least efficient strategy, with a lower enrollment rate (12.97) (Table 2).

#### 3.2.2. Retention

**Figure 3:**
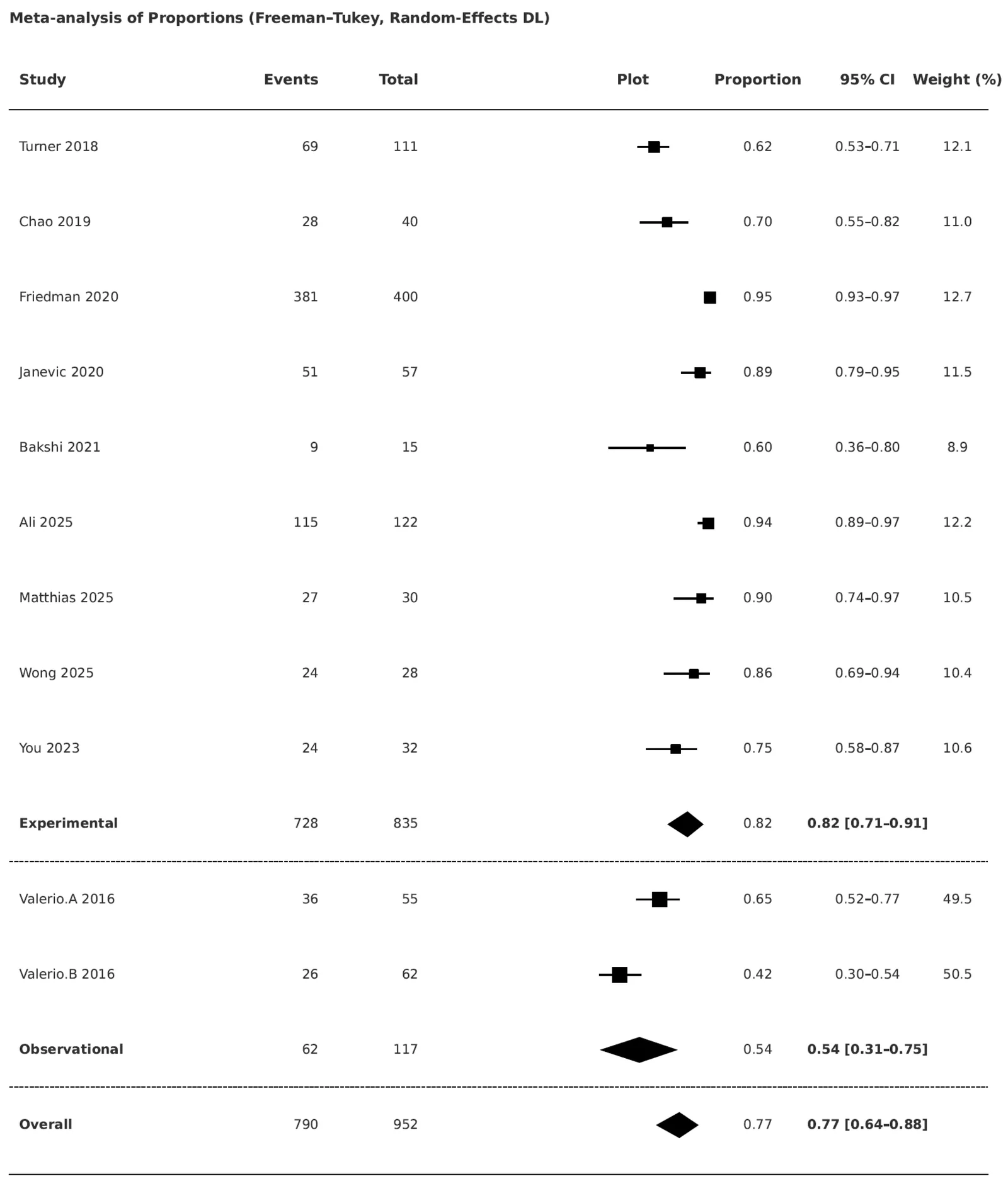
Forest plot of retention rate per study and per design [23,24,26,27,31,33,34,35,36,59].

The proportion of the subjects who completed the trial compared to the enrolled subjects was analyzed as the retention rate in the included studies with more than 75% of the sample as a disadvantaged population. The general retention rate of the studies included was 77% (95% CI 64–88%). The retention rate also did not differ between experimental (82%, 95% CI 71–91%) and observational (54%, 95% CI 31–75%) studies (Figure 3).

A sensitivity analysis showed that those studies with 100% (88.77%, 95% CI 64.28–100%) and 75% (69.22%, 95% CI 42.14–90.73%) of disadvantaged sample did not differ regarding the retention rate (Supplementary Material Figure S2).

In our included studies we mainly detected two retention methods: compensation and reminders. The two methods of retention did not differ regarding the retention rate; compensating the subjects yielded a retention of 76.04% (95% CI 55.71–91.88%), and sending constant reminders yielded a retention of 89.62% (60.43–100%) (Supplementary Material Figure S3). Moreover, when analyzing the retention rate per recruitment strategy, we identified that the studies that used medical records (89.98%) had higher retention than the ones that used snowball (65.5%) and purposive (41.9%) recruitment methods.

### 3.3. Barriers and Facilitators for Clinical Trial Recruitment and Retention

The barriers and facilitators for the recruitment were identified during the extraction and are reported in Table 3. Among all the topics identified, the relationship between the subject and the research staff, clinicians’ engagement in referring and informing the patients about research studies, and the accessibility of the visits (remote visits and follow-ups) were shown to interfere in the recruitment of disadvantaged samples.

### 3.4. Qualitative Analysis

We identified two qualitative studies [30,57] that included 35 subjects (most of them Hispanic and unemployed). After inductive thematic analysis from the interview’s raw quotations, we identified 2 thematic patterns: (1) Patient’s values promoting trial participation: dissatisfaction with the available treatments, desire to reduce pain medications, self-perception of illiteracy on chronic pain, and the belief that interacting with other chronic pain patients and the research team will have a positive influence on their health. (2) Patient’s values affecting trial participation: transportation expenses and inconveniences, changes in pain intensity across days, and the presence of competing activities (family, work, etc.). These converged quantitatively with high-enrollment strategies (community-leader engagement, cultural/language tailoring) and high-retention approaches (compensation to offset costs, flexible reminders). No qualitative–quantitative divergence was observed.

### 3.5. Risk of Bias and Certainty of the Evidence

**Figure 4:**
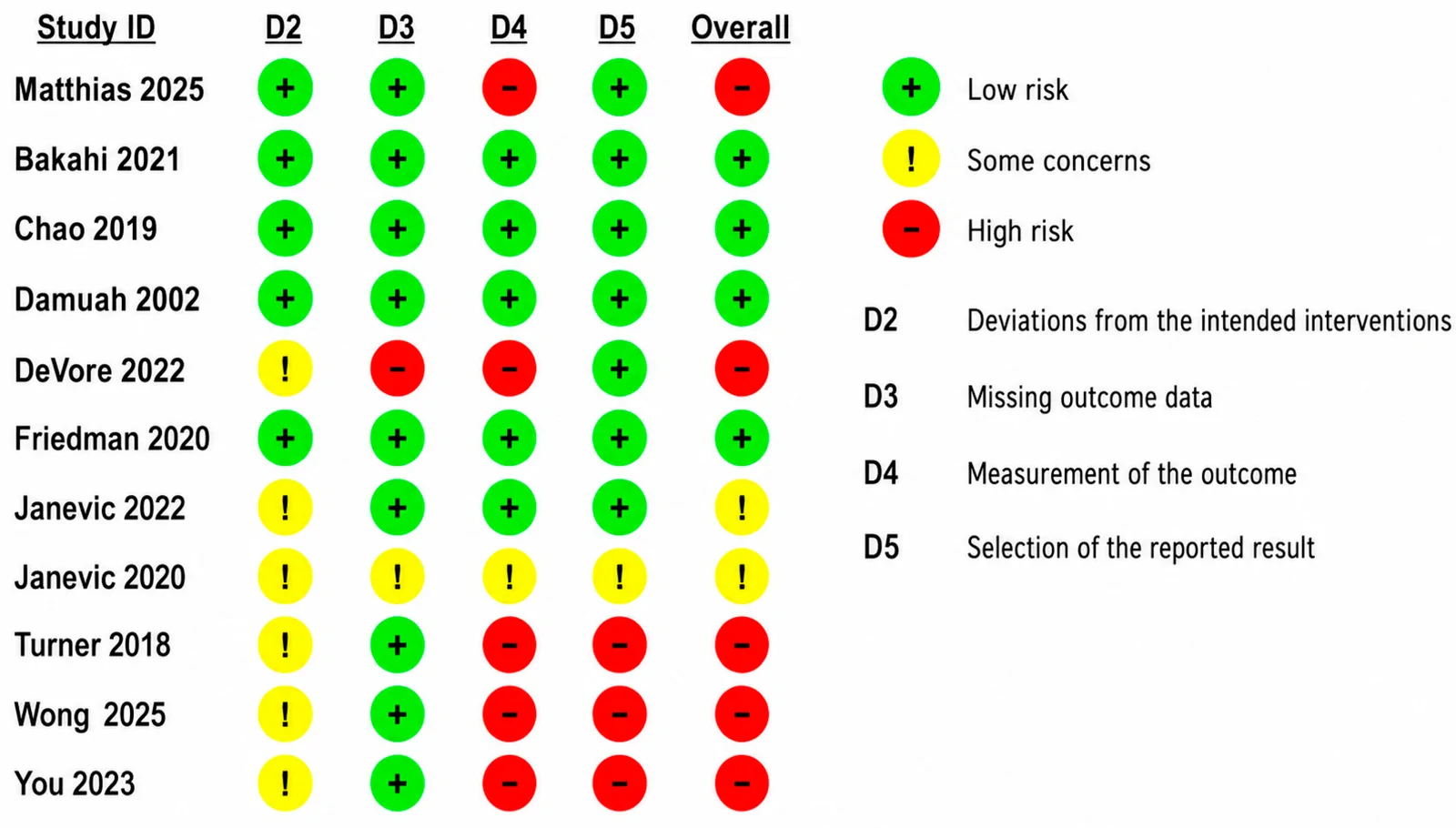
Risk of bias assessment (experimental studies) [23,24,25,26,27,31,34,35,36,55,56].

Using the adapted RoB 2.0 tool, different levels of risk of bias were identified across the included studies. Outcome measurement showed the greatest risk of bias among the evaluated domains. The detailed risk of bias assessment of the included RCTs is represented in Figure 4.

Using the adapted NOS for the purpose of the review and the type of analysis, we assessed the risk of bias of the included observational studies. The studies have shown a high risk of bias mainly in the selection and comparability domains. On the other hand, the qualitative evidence was rated as having a low risk of bias, with some concerns in the domain of validity regarding the prior relationship between researcher and participant. A detailed assessment of each domain is provided in Table 4.

Furthermore, we judged the certainty of the evidence of the meta-analyses for recruitment as having low certainty, and that for retention as having moderate certainty. We started the evaluation from a high level of certainty since all studies were of prospective design. Reasons for downgrading the evidence levels included imprecision (wide CI) and inconsistency (high heterogeneity). The summary of findings is presented in Table 5.

## 4. Discussion

### 4.1. Main Findings

This review aimed to assess the most effective methods to recruit disadvantaged populations to pain studies. Recruitment in the pain field is already reportedly difficult [60], and especially when targeting disadvantaged populations. However, our review conveys that, when recruited, disadvantaged populations in pain appear to be significantly reliable, exhibiting high retention rates throughout studies, with no discrepancy between observational and experimental studies. Nonetheless, our results convey a discrepancy between enrollment and retention rates in these populations; given they are a narrower population and need to yield a significant time commitment to studies, their enrollment rates in studies are low, but once they are enrolled, the access to treatment and higher expectations of its effectiveness induce higher retention rates, emphasizing the reliability of this population.

To our knowledge, this is the first review to combine quantitative and qualitative evidence to evaluate recruitment and retention of ethnically diverse and socioeconomically disadvantaged populations in pain research. It is also the first to quantitatively compare recruitment and retention performance across different recruitment and retention methods using meta-analyses of proportions. By integrating pooled estimates with thematic synthesis, this review provides both quantitative evidence of strategy effectiveness and contextual insights into the factors influencing participation.

Rather than only describing barriers and facilitators, this review extends existing recruitment and equity frameworks by linking implementation strategies with their observed effectiveness. This provides an evidence-informed approach to guide the selection of recruitment and retention strategies in future pain research involving ethnically diverse and socioeconomically disadvantaged populations.

### 4.2. The Challenge of Recruitment of Disadvantaged Populations in Pain Research

The substantial variability observed in enrollment rates across studies likely reflects differences in pain conditions, recruitment settings, study design, and healthcare contexts. Together, these factors influence the barriers faced by underrepresented populations and may explain the heterogeneity in enrollment estimates. These findings reinforce the need to tailor recruitment strategies to the target population and study setting rather than assuming a universally effective approach.

This comprehensive review aimed to assess the recruitment and retention efficiency and strategies in clinical studies for disadvantaged pain populations. Our results showed that even though our studies included 75% of disadvantaged populations in their sample size, the recruitment rates were low and difficult to accomplish. Two factors that play an important role in this finding are: the characteristics of the disadvantaged populations and of the subjects with pain.

Disadvantaged populations usually have low socioeconomic status compared to the white population [61,62]. This can be accompanied by financial barriers and low education levels [63], which leads to decreased access to the health systems and decreased exposure to treatment options, including medical research [64]. Moreover, these populations might present with low socioeconomic status and as such lack job flexible schedules that could accommodate the schedule of clinical trials, rendering them unable to adhere to the protocols’ visits [63].

The U.S. healthcare system’s historical mistrust, as exemplified by the Tuskegee Study [65], combined with low health literacy and language barriers (e.g., Spanish-only Hispanic populations), undermines the trust essential for the recruitment of ethnically diverse groups. Additionally, research funding is concentrated in states like California, New York, and Massachusetts [66], where white populations predominate (e.g., Massachusetts: 80.6% white vs. 9% Black, 12.4% Hispanic per the 2020 Census) [67], potentially limiting exposure to and experience with disadvantaged populations and contributing to under-recruitment.

Regarding the chronic pain population (the majority of the studies identified), according to the 2016 National Health Interview Survey (NHIS) data, “20.4% (50.0 million) of U.S. adults had chronic pain and 8.0% of U.S. adults (19.6 million) had high-impact chronic pain” [68] (defined as chronic pain that daily or mostly daily limited life or work activities for about 6 months [68]), with higher prevalence in women, white non-Hispanic people, people of advanced age, people previously but not currently employed, and adults living in poverty [68]. Moreover, patients with chronic pain are more likely to present disabling pain that can restrict movement and daily life activities, increased dependence on opioids and its side effects, increased prevalence of psychiatric conditions such as depression or anxiety and increased polypharmacy [68,69]. These characteristics may hinder recruitment and retention. For example, the presence of pharmacological interactions and severe depression may constitute an exclusion criterion for participation in studies. Also, disabling pain, advanced age, and pharmacological side effects can decrease adherence to studies as well as transportation, and the nature of the study can become a strain on the subject. Moreover, the time allocated to these studies can also be a barrier.

Nonetheless, despite the aforementioned barriers, our results showed that once this population is recruited, they have high retention rates. This could be explained by the following reasons: first, the lack of access to health care of this population, and the fact that research studies may be the only way for patients without insurance to receive any type of treatment. Second, due to the characteristics of low socioeconomic status in these populations, the extra income they can receive by way of compensation for their participation in such research may be an incentive to stay for the duration of the trials. Third is the expectations the patients have regarding the trial, including the belief that the research intervention may decrease their pain levels. And finally, if the research team was able to provide a safe, inclusive, and welcoming and friendly space, it could have improved the trust and the relationship between the research team and the participants, leading to increased adherence [70].

### 4.3. Evidence-Based Recruitment Strategies

Our results convey that snowball sampling yielded the highest enrollment rate of disadvantaged populations in different pain studies. This is consistent with other studies evaluating the recruitment of ethnic minorities for colorectal cancer trials [71]. As discussed by Rodriguez et al. (2006), referral from a known member in disadvantaged communities establishes trust and eliminates concerns around clinical trials [72]. Establishing trust with marginalized groups is a priority considering the distrust of these communities in research due to its egregious historical legacy with disadvantaged populations [73], and several observational studies have proven the feasibility of this method for the recruitment and retention rates of these populations [59,74]. In fact, when comparing it to other non-probability methods of sampling, snowball sampling has been proven to be the most effective method of recruiting marginalized populations to pain studies [59].

Another recruitment method used in this review’s pooled studies is purposive recruitment [59]. It is characterized by an optimized, community-based approach that identifies individuals within a sample that might fit a study’s profile [75]. One way of conducting this non-probabilistic sampling method is through the engagement of community leaders and champions within historically excluded populations [59], once again reinforcing the impact of trust among disadvantaged communities and research. Interestingly, some studies have proposed a combined recruitment method, purposive–snowball, in which leaders and influencers in disadvantaged communities can identify possible interested and eligible subjects for pain studies [75]. Moreover, although snowball sampling and purposive recruitment methods may lead to sample bias during the recruitment process, considering that historically marginalized individual enrollment in clinical studies has shown recruitment rates as low as 2–16%, this bias then becomes necessary in order to appropriately represent these subjects in clinical research [67,75].

Referrals from clinicians appear to be another reliable, evidence-based recruitment method for the enrollment of disadvantaged individuals in pain studies [25]. The effectiveness of this strategy is based on the level of trust between possible participants and their physicians, which can influence a myriad of aspects from a subject’s willingness to participate, to their adherence to treatment protocol [76]. Nonetheless, even though referrals may be depicted as an effective recruitment strategy, it depends largely on the interest of physicians in the research being conducted. A recent study evaluating recruitment methods for disadvantaged individuals with fibromyalgia showed that only 10% of subjects were told about research studies by their physicians [32]. This conveys a lack of physician immersion in clinical research, posing a challenge to the optimization of this approach, and raising questions on how to further integrate clinical research in the healthcare community.

### 4.4. Barriers and Facilitators of Recruitment and Retention

We identified barriers and facilitators for the recruitment process that we divided into the following three categories:

#### 4.4.1. Access to Information About Clinical Trials

Advertisement for clinical trials has been done in many different ways, from local and national newspaper ads—which have shown not to be as effective as researchers would want [77]—to flyers in authorized public places, direct mail letters, among others. Nowadays, in the digital era, online recruitment strategies have proven to be effective in increasing recruitment rates, but they can be more expensive [78]. Moreover, information about ongoing private and funded trials can be found in the NIH database ClinicalTrials.gov [79], and on different websites focused on recruiting people for research studies. One important concern is that all strategies must be approved by an institutional review board before application. In other words, the information about a clinical trial could be a click away and still there are many people who are not aware of them. One important link that we recognized with our review was the importance of the physician figure. For white women, having a primary care physician was shown to be a facilitator, leading us to the conclusion that a clinical referral was done. However, on the other hand, a barrier identified was the reluctance of clinicians to refer to studies. This may be due to the gap between clinical work and clinical research and the fact that physicians, due to lack of time as well as incentive, may focus more on clinical work than on research [76]. However, the trust between physicians and researchers also plays an important role. For example, the results of a survey that assessed physician’s attitudes and beliefs regarding patient recruitment, more specifically in disadvantaged populations, reported that “African American physicians and those with the highest proportion of minority patients in their practices had the lowest levels of trust” [80] in medical research. Therefore, there is a need to directly share this information and create good relationships not only with possible subjects but also with physicians that treat the target population (this could be done by email, presentations, personal visits and calls).

Nonetheless, this will only apply to subjects that have access to healthcare, which unfortunately is not the case for many disadvantaged populations in the US due to a lack of health insurance and other access barriers [81,82]. This may explain why, for black women, health care figures were not effective facilitators, but mass emails were. Thus, continuing these types of practices and other community-targeted strategies may be helpful to recruit this population.

#### 4.4.2. Characteristics of the Study

The study design may not only be planned based on the scientific approach but also considering its feasibility and ways to decrease the time burden for participants. In the recruitment process, stringent inclusion and exclusion criteria will become a barrier, as it narrows the pool of the general population and makes it harder to find individuals that fit all the criteria. As for facilitators, the ability of a study to be hybrid (in-person and partially remote) and/or with follow-ups done by mail or phone calls provides an incentive for the participants, as it decreases the burden of time and transportation and subjects are more inclined to want to participate. Moreover, a flexible schedule and the absence of time restrictions for the consent and enrollment process provide more options for the subjects and possibilities to arrange their time to be able to participate fully in the trial.

### 4.5. Characteristics of the Population

Age, specifically older age, can be considered a barrier and facilitator. On one hand, with older age there is higher frailty [83] and more comorbidities that could lead to undesired side effects and mobility impairment [84]. This translates to issues with transportation and this may hinder the possibility of an older subject participating in an in-person study [85]. On the other hand, the older population is often retired and has more free time to participate in different studies, in contrast to the working population. Advanced age can also be a barrier regarding new technologies. There is a trend in clinical research to develop online tools to access and study patients. Commonly, older people present a higher resistance to learning and lower familiarity with such technological and online tools when compared with younger subjects.

Furthermore, financial status can also be a facilitator, regardless of age. Having an unstable financial status may increase recruitment, as this population may be more inclined to participate in studies that give economic incentives.

In addition, language barriers can hinder the understanding of the trial itself, as well as the assessment during the trial and the relationship between the subject and the researcher. Thus, it comes as no surprise that a subject’s knowledge of English is considered a facilitator. This must be considered beforehand, during the planning of the study; otherwise, the inability to include non-English speakers will make it harder to recruit disadvantaged populations such as Hispanics/Latinos.

### 4.6. Retention of These Populations in Pain Studies

As we previously mentioned, the overall retention was high (76.74%) and most of the studies used compensation strategies and reminders. Furthermore, medical records are a source of reliable patients; however, we need to target institutions with a high proportion of disadvantaged populations.

This pattern of low enrollment (37%) but high retention (77%) among ethnically diverse and socioeconomically disadvantaged populations indicates that structural access barriers—rather than low interest or poor adherence—primarily limit equitable pain research participation. Once enrolled, these groups show strong engagement comparable to other populations, emphasizing the need to prioritize community-engaged recruitment strategies over retention interventions alone.

As one of the successful retention strategies was the use of reminder calls, this method could be improved by the use of automated messages, which could reach all participants in the event that they cannot be reached over the phone. Using electronic data collectors, mainly for information that subjects need to collect in their homes, should be encouraged, with a special interest in those that can send reminders of follow-up visits. The use of a study coordinator that can keep clear track of the subjects, from recruitment to the conclusion of the study, could help retention, as this person would be in charge of the reminder calls and following up with every subject and each individual’s special needs [86].

It was also seen that incentives play an important role in the retention of subjects. The monetary incentive, though helpful for low-income populations, may not be their main concern, as disadvantaged populations have an increased need for help caring for their dependents; therefore, providing childcare assistance could help retention. Transportation is important for disadvantaged populations, who mainly depend on public transportation for their mobilization, or even for those who have access to vehicles but have higher distance to travel [85]. In the case of pain populations, longer commuting hours could decrease their retention.

Another good way to increase retention is providing educational material to the study subject regarding the importance of attending every study visit, and also using incentives such as gift bags at the end of the study to promote attendance to the final visit [87].

Moreover, successful rapport of the investigator with the study subject can help retention. If there is a good relationship between them and the subjects feel that their participation is meaningful and helpful, it may be more likely that the subject will continue participating in the study. Therefore, having a more diverse and representative environment, the option to communicate in their native language, and the use of respectful and inclusive language could provide a safe environment in which disadvantaged populations feel more comfortable.

### 4.7. Recommendations

**Figure 5:**
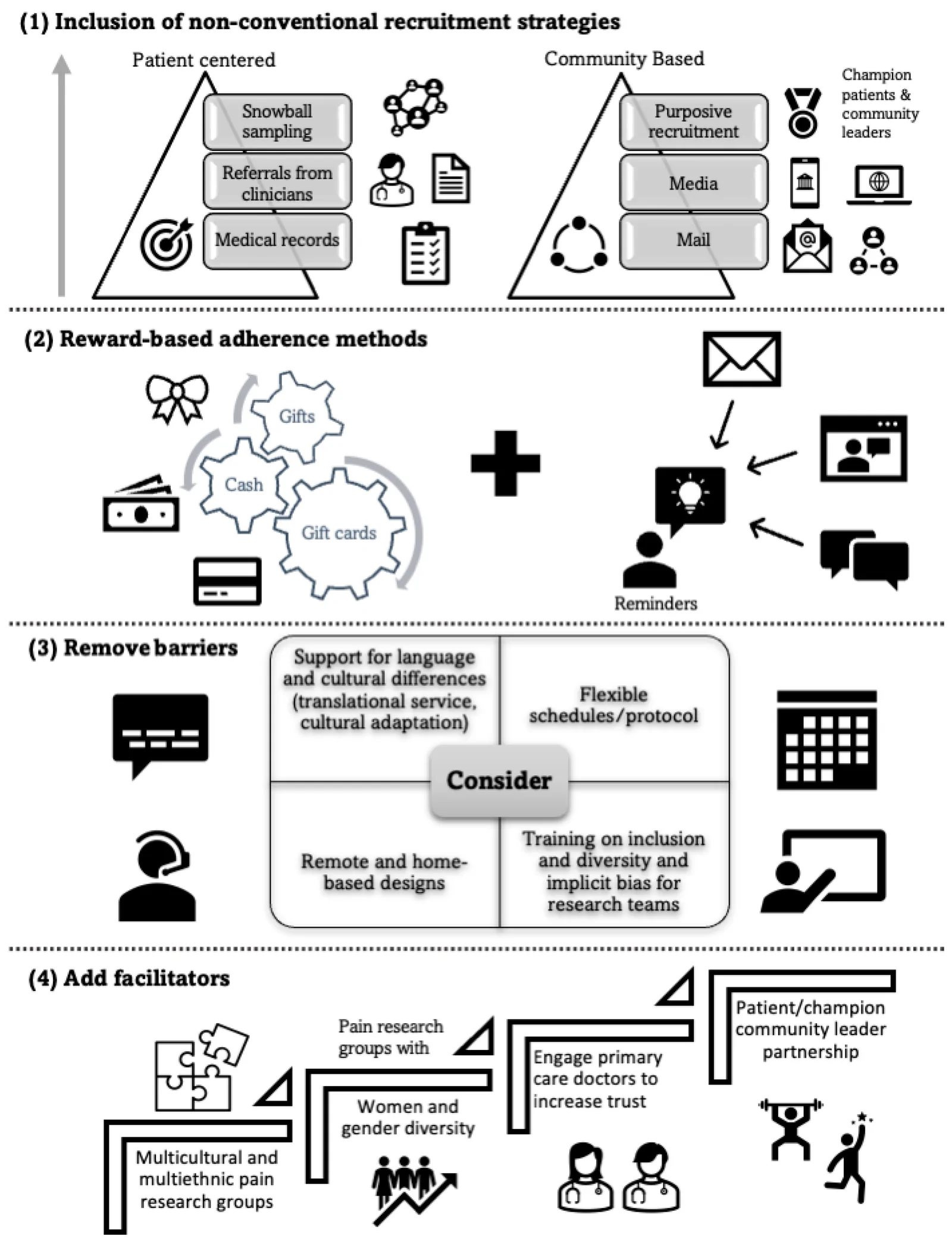
Evidence-based recommendations for recruitment and retention of disadvantaged populations in pain studies.

Our results showed that different recruitment strategies may be useful depending on the goals in conducting a research study. Some strategies were shown to be more efficient in enrolling disadvantaged populations, such as purposive and snowball methods, while others were shown to bring more reliable and constant participants to the studies, like medical records. Additionally, regarding the retention methods, both approaches analyzed (compensation and reminders) yielded higher retention rates, without meaningful differences between them. These results may convey that the best approach in developing a clinical trial for chronic pain populations is to mix specific methods to recruit disadvantaged populations while encouraging them to complete the entire protocol. The strategy of mixing recruitment and retention strategies must allow the possibility of taking advantage of the better features of each strategy while protecting against their flaws. Enrollment and retention flowcharts were poorly reported in most studies, omitting key metrics such as numbers contacted, screened, eligible, and lost at each stage, the number of completers, as well as the proportion of enrolled participants meeting the ≥75% disadvantaged threshold. This limits precise yield calculations and strategy comparisons. Future primary studies should adopt standardized CONSORT-style flowcharts disaggregating by ethnicity/socioeconomic status to enable robust syntheses and evidence-based improvements. Additionally, if it is possible, these numbers should be reported per recruitment and retention strategy. Our recommendations are summarized in Figure 5.

### 4.8. Limitations

Although this review and meta-analysis yielded consistent results, it does have some limitations, the main one being the definition of underrepresentation. Different chronic pain conditions may have different epidemiological patterns, and thus different disadvantaged populations. Moreover, although a large group of studies focus on ethnical minorities as disadvantaged population in pain, it is worth noting that pooling them together to assess recruitment strategy effectiveness can be misleading, and lead to ineffective methods of recruitment, through a “one-size-fits-all” approach.

Moreover, restricting inclusion to studies in which at least 75% of participants were from underrepresented populations may limit the generalizability of these findings to more heterogeneous clinical trial populations. However, this criterion ensured that the identified recruitment barriers, facilitators, and strategies primarily reflected the experiences of the populations of interest.

Moreover, the predominance of U.S.-based studies (17/18) limits generalizability to other healthcare systems and social contexts. U.S. findings reflect fragmented insurance structures, which may not translate directly to single-payer systems (e.g., Europe, Canada) or low-resource settings where structural access barriers differ. Although core facilitators like community engagement and cultural tailoring likely remain relevant, validation studies in diverse international contexts are needed to confirm strategy effectiveness across varying healthcare and socioeconomic environments.

Qualitative findings from only two U.S.-based studies offered valuable insights into patient values but constitute limited evidence. Themes promoting participation (treatment dissatisfaction, pain literacy gaps) align with high-enrollment strategies like community engagement and cultural tailoring, while barriers (transportation costs, fluctuating pain, competing demands) converge with effective retention solutions (compensation, flexible scheduling). These should not be overgeneralized beyond similar chronic pain patients within similar healthcare systems without confirmation from larger, diverse qualitative datasets.

The integration of quantitative and qualitative evidence represents an important strength of this review. While the quantitative analyses identified which recruitment and retention strategies were associated with higher participation, the qualitative findings helped explain why these strategies were effective by identifying participant-perceived barriers and facilitators, including trust, culturally tailored communication, transportation, and flexible study procedures. Together, these complementary approaches provide a more comprehensive understanding of trial participation and may better inform the design of future equitable pain research.

Pooled meta-analyses provide informative benchmarks but remain exploratory due to heterogeneity from study design (observational > experimental enrollment), pain type (chronic dominance across distinct conditions with varying underrepresentation rates), and settings (U.S. healthcare). Pooling recruitment strategies from different chronic pain conditions can yield ambiguous results. Furthermore, although including both acute and chronic pain populations increased the comprehensiveness of this review, recruitment and retention dynamics may differ between these populations and should be evaluated separately in future studies whenever sufficient evidence is available. Validation via meta-regression or primary studies is required when larger evidence bases permit. Furthermore, pooling ethnically diverse groups (e.g., Black, Hispanic/Latino, Indigenous) and heterogeneous SES profiles into a single “underrepresented” category risks oversimplifying within- and between-group differences, as structural barriers and facilitators may vary substantially by specific ethnic identity and SES marker. For example, income-based disadvantage may interact differently with pain-related stigma than insurance-based exclusion. Our pooled estimates thus represent average effects across broad categories rather than group-specific impacts, and may mask recruitment and retention barriers that are unique to specific ethnic groups or socioeconomic contexts; given this, readers should interpret results cautiously when extrapolating to particular populations. Future studies should standardize and disaggregate recruitment/retention metrics by specific ethnic groups and SES indicators (e.g., income quartiles, validated deprivation indices) to enable more granular syntheses.

Thus, it is important for researchers to keep in mind different recruitment approaches for different historically marginalized populations, as strategies that work for one group might not necessarily work for another.

## 5. Conclusions

Our review synthesizes the recruitment strategies reported in the literature for disadvantaged populations in pain. The recruitment and retention of these populations is challenging and a crucial part of research. Our study showed a low enrollment rate but generally high retention rates among the included studies, suggesting that barriers to participation may occur primarily before enrollment rather than after recruitment. Diverse recruitment and retention strategies were identified, providing initial evidence for their advantages and drawbacks. Incomplete reporting of enrollment flowcharts and disadvantaged population proportions hinders strategy evaluation and meta-synthesis. Standardized CONSORT-adapted diagrams specifying screening-to-enrollment yields by ethnicity/socioeconomic status are essential for advancing equitable pain research participation.

Future studies reporting their strategies and future reviews analyzing these studies are necessary to gather data and build knowledge in the best way to combine and take advantage of each strategy based on specific aims.

## Figures and Tables

**Table 1:** Characteristics of included studies.

Author	Country	Design	Target Population	Sample Size	Pain Intensity	Participants	Recruitment	Retention
Ali et al. (2025), [33]	US	Observational	Chronic pain in Sickle cell disease	122	Not reported	Mean age: 33.4 (10.6) Female: 83 (68%) *Race* African American: 113 (93%)	Hematology clinics both locally and statewide. Integrated Data Repository List. Community events and involvement statewide. Referrals from another sickle cell study. Targeted outreach to community organizations nationwide, and implementing social media campaigns and influencers.	Not reported
Bakshi et al. (2021) [23]	US	Experimental	Teenagers with chronic pain in Sickle cell disease	15	61.9(58.5–68.4)	Median age: 16 (14–17) Female: 8 (53.3%) *Race* African American: 12 (80%) More than one race: 3 (20%) *Ethnicity* Non-Hispanic or Latino 13 (86.7%) Hispanic or Latino 2 (13.3%)	Medical records Community sites	Financial compensation
Chao et al. (2019) [24]	US	Experimental	Painful diabetic neuropathy	40	5.17	Average age: 59.03 Female: 20 (50%) *Race/ethnicity* African American: 8 (20%) Asian: 4 (10%) Latino: 20 (50%) Non-Latino white: 7 (17.5%) Other: 1 (2.5%)	Media (Posting flyers in Chinese, English, and Spanish) Medical records	Financial compensation
Damush et al. (2002) [25]	US	Experimental	Patients with acute low back pain	211	8.1 (2.5)	Average age: 46 (19–83) Female: 154 (73%) *Race/Ethnicity* African American: 127 (60%) Hispanic: 2 (1%) *Socioeconomic status:* Insufficient income to meet their needs (19%) Income was enough to just make ends meet (62%) Income was not enough to make ends meet (15%)	Clinician referral	Reminders Financial compensation
DeVon et al. (2022) [55]	US	Experimental	Stable angina	24	Intervention group 3.81 (1.99) Control group 3.42 (2.56)	Females: 15 (63%). *Ethnicity* White 2 (8%), Black/African American 11 (46%), Hispanic participants 9 (38%), other 2 (8%). *Socioeconomic status* Lower income (96%; annual income <US$50,000).	From the Cardiology clinic	Not reported
Friedman et al. (2020) [26]	US	Experimental	Patients with chest pain admitted to a medical center	400	Not reported	Average age: 57 Female: 252 (63%) *Race* Asian: 18(4.5%) Black: 143(35.8%) Hispanic: 215(53.8%) Multiracial: 6(1.5%) White 18(4.5%)	Medical records Email Text	Reminders
Janevic et al. (2020) [27]	US	Experimental	Older African American with chronic musculoskeletal pain	57	5.2 (1.8)	Average age: 70.4 (6.8) Female: 45 (88%) African American: 50 (98%)	Media (flyers at community locations serving older adults) Medical records (health research volunteer registry of African American older adults)	Compensation (gift card)
Janevic et al. (2022) [56]	US	Experimental	Older African American adults with chronic musculoskeletal pain	46	6.2 (1.7)	89% female, 100% identified as African American or multiracial 24% reported difficulty paying bills each month.	Medical records (health research volunteer registry of African American older adults) Word of mouth at community locations for older adults (list of interested volunteers)	Not reported
Joyce et al. (2022) [57]	US	Qualitative	Adults with low back pain	26	7.2 (NRS)	female (69%), and black (77%), 16 <$40,000/year	Patients were asked at the end of an intervention. For this intervention they were recruited from a medical center and community health centers.	Not reported
Masese et al. (2021) [28]	US	Observational	Sickle cell disease	2432	Not reported	Average age: 28.1(7.9) Female: 1369 (56.9%) Black or African American: 2237 (95.3%) Multiracial: 80 (3.4%) Other: 30 (1.3%)	Community sites Targeted phone calls Community events	Participant compensation
Matthias et al. (2025) [34]	US	Experimental	Musculoskeletal pain	30	6.3 (1.55)	Average age: 60.6 (13.6) Female: 21 (70.0%) Black or African American: 28 (93.3%)	Electronic medical records Recruitment letters	Not reported
Nissen et al. (2022) [58]	Norway	Observational	Syrian formal refugee in Norway	902	Not reported	64.5% of participants were men, 100% Syrian refugees.	Postal invitation, data from national registry	One postal or telephone reminder to all non-responders
Turner et al. (2018) [31]	US	Experimental	Low-income and Hispanic patients	111	Not reported	Average age: 56.5 Female: 61(54.9%) Hispanic: 87 (78.4%) Non-Hispanic white: 14 (12.6%) Non-Hispanic black: 10 (9%)	Medical records	Not reported
Turner et al. (2020) [30]	US	Qualitative	Low-income and Hispanic patients with chronic non-cancer pain	18	Not reported	Average age: 57 Female: 7 (39%) Hispanic: 12 (67%) Non-Hispanic white: 4 (22%) Non-Hispanic black: 2 (11%)	Medical records	Instructors had chronic pain conditions, group sessions for team building, sessions with instructors and individual support.
Valerio et al. (2016)-A [59]	US	Observational	Hispanic communities with chronic non-cancer pain	55	Not reported	Average age: 58 Female: 38 (69%) Hispanic 48 (87%) Non-Hispanic white (13%)	Snowball	Not reported
Valerio et al. (2016)-B [59]	US	Observational	Hispanic communities with chronic non-cancer pain	62	Not reported	Average age: 57 Female: 40 (65%) Hispanic: 45 (73%) Non-Hispanic white: 17 (27%)	Purposive strategy and convenience sampling	Not reported
Wong et al. (2025) [35]	US	Experimental	Black and Hispanic/Latino people with peripheral neuropathy	28	7.2 (2.1)	Age: 58.6 (9.4) Female: 20 (71%) African American: 16 (57%). Hispanic: 12 (43%).	Pre-trial online recruitment survey strategy	Not reported
You et al. (2023) [36]	US	Experimental	Chronic multisite musculoskeletal pain	32	Not reported	Age: 100% older than 65 years old Female: 21 (65.6%) African American: 17 (53.1%)	Community targeted mass mailings	Not reported

**Table 2:** Enrollment rate per recruitment strategy.

Recruitment Strategy	Enrollment Rate (%)
*State-wide specific disease clinics (Sickle cell clinic)* Ali, [33]	89.00
*Purposive community leader strategy* Valerio. B, [59]	87.30
*Snowball* Valerio. A, [59]	82.20
*Doctor referral* Damush, [25]	37.19
*Social media and influencers* Ali, [33]	10.10
*Medical records* Turner, [31] Ali, [33]	12.97 0.90

Note: Recruitment strategies are written in italics.

**Table 3:** Barriers and facilitators.

Author	Identified Barriers	Identified Facilitators
Ali et al. (2025), [33]	Personal factors Trouble adhering to specific data entry time windows Overwhelming pain at home or before or after a doctor’s visit	Not reported
Bakshi et al. (2021), [23]	Stringent inclusion and exclusion criteria	Not reported
Chao et al. (2019), [24]	Not reported	Not reported
Damush et al. (2002), [25]	Not reported	Target older and financially unstable groups
DeVon (2022), [55]	Nature of the intervention (Acupuncture)	Recruiting patients during medical care. Adaptation of the intervention protocol for the target population following standard recommendations.
Friedman et al. (2020), [26]	Not reported	Having a Primary Care Physician and English as native language
Janevic et al. (2020), [27]	Older age	Partially remote protocol
Janevic et al. (2022), [56]	Older age Not enough access to technology	Remote protocol Focus groups include similar population of patients and of health care providers to validate and tailor the intervention content. Cultural adaptation of the material (for the African American community).
Joyce et al. (2022), [57]	Distance/transportation and costs: These were related in some cases, including the costs for continuity after the trial (which was free) was completed. Credibility of the self-care book. Readability and social isolation: Difficulties understanding and interpreting the written material due to literacy, engagement, or familiarity with English. Participants reported they would have liked to discuss the book with others who had read it or practiced the content before.	Communal support and camaraderie: Between participants because of shared diagnosis, which they find stigmatizing if not taken seriously by outsiders. Empowerment through knowledge: Understanding their body allows the patients to discover which activities they could do and which strategies to use to have little or no pain.
Masese et al. (2021), [28]	Clinic visits: disruption of clinic workflow; limited research personnel to complete study procedures with all eligible participants; lack of space or rooms in the clinic for recruitment activities; and competing SCD-related research studies. Affiliated sites: difficulty in obtaining SCD diagnosis confirmation. Community events: same group of individuals attended most of the community events; inability to obtain sickle cell disease confirmation.	Inpatients: absence of time restrictions for the consent and enrollment process
Matthias et al. (2025), [34]	Not reported	Not reported
Nissen et al. (2022), [58]	Potential negative feeling during the survey (including questions regarding traumatic events). Completely unguided.	Use of an official registry for a minority population. One postal or telephone reminder to all non-responders. Instructions for how to deal with issues in case negative feelings arose during the survey.
Turner et al. (2018), [31]	Not reported	Not reported
Turner et al. (2020), [30]	Availability of the sample, age of instructors, distance	Having a specific supervisor that followed up each patient during the whole trial and instructors that also had chronic pain
Valerio et al. (2016)-A, [59]	Not reported	Not reported
Valerio et al. (2016)-B, [59]	Not reported	Not reported
Wong et al. (2025), [35]	Technology-related difficulties using the online platform	Mixture of in-person and online activities
You et al. (2023), [36]	Not reported	Not reported

**Table 4:** Quality assessment for observational and qualitative studies.

Observational Studies			
Study ID	Selection	Comparability	Exposure
Ali (2025), [33]	★★★☆☆	☆☆	★★☆
Nissen (2022), [58]	★★★★☆	★☆	★★☆
Masese (2021), [28]	★★☆☆☆	☆☆	★☆☆
Valerio (2016)-A, [59]	★★☆☆☆	☆☆	★★★
Valerio (2016)-B, [59]	★★☆☆☆	☆☆	★★★
**Qualitative studies**			
Study ID	Validity	Interpretability	Aplicability
Joyce (2022), [57]	6 out of 6	2 out of 3	1 out of 1
Turner (2020), [30]	5 out of 6	3 out of 3	1 out of 1

Note: Stars refer to the total number of points possible to achieve in each domain, with black stars referring to scored points according to the criteria of the Newcastle– Ottawa Quality Assessment Scale (NOS) (non- RCTs).

**Table 5:** Summary of findings of GRADE evaluation.

Disadvantaged Population				
Population: Disadvantaged populations Exposure: Participation in pain studies				
**Outcomes in proportion**	Anticipated absolute effects (95% CI)		№ of participants (Studies)	Certainty of the evidence (GRADE)
	Proportion	95% CI		
**Enrolment**	0.37	0.18 to 0.58	2032 individuals (7 studies)	⨁⨁◯◯ LOW ^a,b,c,d,e,f^
**Retention**	0.77	0.64 to 0.88	952 patients (10 studies)	⨁⨁⨁◯ MODERATE ^a,b,c,d,e,g^
CI: Confidence interval				
Explanations The certainty rating started from high since we included studies of prospective design and that is the ideal design for recruitment. Low risk of bias: N/A. High inconsistency was present in the meta-analyses with an I^2^ > 60%, so we decided to downgrade the evidence. Indirectness was not present since the evidence came from the outcomes of interest. Publication bias: N/A. Imprecision was present, since the range of the CI exceeded the 50% value of the estimate. Imprecision was not present.				
**GRADE Working Group grades of evidence** **High certainty:** We are very confident that the true effect lies close to that of the estimate of the effect. **Moderate certainty:** We are moderately confident in the effect estimate: The true effect is likely to be close to the estimate of the effect, but there is a possibility that it is substantially different. **Low certainty:** Our confidence in the effect estimate is limited: The true effect may be substantially different from the estimate of the effect. **Very low certainty:** We have very little confidence in the effect estimate: The true effect is likely to be substantially different from the estimate of the effect.				

Note: Circles refers to the possible punctuation of evidence certainty, from those each plus sign refer to one attributed point (very low: 1, low: 2, moderate: 3, and high: 4).

## Data Availability

No new data were created or analyzed in this study, and all data used in this review paper is available to the public.

## Supplementary Materials

The following supporting information can be downloaded at: https://www.mdpi.com/article/10.3390/ijerph23081091/s1, File S1: Authorship Note of Equity in Pain Research Working Group (EPR-WG); File S2: PRISMA checklist; File S3: Search strategy; Table S1: List of excluded studies with reasons; Figure S1: Sensitivity analysis of enrolment rate; Figure S2: Sensitivity analysis of retention rate; Figure S3: Forest plot of the retention strategies.
