# Supplementary material for: Improving Inclusion of Ethnically Diverse and Socioeconomically Disadvantaged Populations in Pain Research: A Comprehensive Review and Evidence-Based Recommendations
Source: Int J Environ Res Public Health. 2026 Aug 21;23(8):1091. doi: 10.3390/ijerph23081091 (PMC13512727; doi:10.3390/ijerph23081091)
Supplement: Supplementary file 1 [file ijerph-23-01091-s001.zip › ijerph-4483608-supplementary.pdf]

Supplementary material for

Improving Inclusion of Ethnically Diverse and Socioeconomically Disadvantaged Populations in Pain Research: A Mixed-Methods Systematic Review and Evidence-Based Recommendations

K Pacheco-Barrios et al.

Equity in Pain Research Working Group (EPR-WG) Collaborators

Supplementary Material File S1. Authorship Note of Equity in Pain Research Working Group (EPR-WG):

This work was conducted on behalf of the Equity in Pain Research Working Group (EPR-WG), a collaborative network dedicated to advancing equity, representation, and inclusion in pain science. All individual authors listed in the manuscript meet full ICMJE criteria for authorship, and the group designation reflects the coordinated effort, shared methodological framework, and interdisciplinary expertise contributed by members of the Working Group. The EPR-WG provided conceptual input, methodological guidance, and critical revision throughout the development of this review. A complete list of contributing members of the Equity in Pain Research Working Group is provided below:

Anna Marduy [1], Karen Vasquez-Avila [1], Ingrid Rebello-Sanchez [1], Joao Parente [1], Luna Vasconcelos Felippe [1], Stefano Giannoni-Luza [1], Roberto M. Machado [1], Danielle C. Pimenta [1], Daniel Lima [1], Elly Pichardo [1], Rafaela Machado Filardi [1], Alejandra Cardenas-Rojas [1], Maria Andrade [1], Wolnei Caumo[2,3].

Affiliations:

1 Neuromodulation Center and Center for Clinical Research Learning, Spaulding Rehabilitation Hospital and Massachusetts General Hospital, Harvard Medical School, Boston, USA.

2 Laboratory of Pain and Neuromodulation, Hospital de Clínicas de Porto Alegre, Porto Alegre, Brazil.

[3] Post-Graduate Program in Medical Sciences, School of Medicine, Universidade Federal do Rio Grande do Sul, Porto Alegre, Brazil.

Supplementary material File S2. PRISMA checklist

Section/topic	#	Checklist item	Page #
**TITLE**			
Title	1	Identify the report as a literature review.	01
**ABSTRACT**			
Structured summary	2	Provide a structured summary including, as applicable: background; objectives; data sources; study eligibility criteria, participants, and interventions; study appraisal and synthesis methods; results; limitations; conclusions and implications of key findings;	02
**INTRODUCTION**			
Rationale	3	Describe the rationale for the review in the context of what is already known about your topic.	03
Objectives	4	Provide an explicit statement of questions being addressed with reference to participants, interventions, comparisons, outcomes, and study design (PICOS).	04
**METHODS**			
Eligibility criteria	5	Specify study characteristics (e.g., PICOS, length of follow-up) and report characteristics (e.g., years considered, language, publication status) used as criteria for eligibility, giving rationale.	04
Information sources	6	Describe all information sources (e.g., databases with dates of coverage) in the search and date last searched.	04
Search	7	Present full electronic search strategy for at least one database, including any limits used, such that it could be repeated.	04
Study selection	8	State the process for selecting studies (i.e., screening, eligibility).	05
Risk of bias in individual studies	9	Describe methods used for assessing risk of bias of individual studies (including specification of whether this was done at the study or outcome level).	05
Risk of bias across studies	10	Specify any assessment of risk of bias that may affect the cumulative evidence (e.g., publication bias, selective reporting within studies).	06

Section/topic	#	Checklist item	Reported on page #
**RESULTS**			
Study selection	11	Give numbers of studies screened, assessed for eligibility, and included in the review, with reasons for exclusions at each stage, ideally with a flow diagram.	7
Study characteristics	12	For each study, present characteristics for which data were extracted (e.g., study size, PICOS, follow-up period) and provide the citations.	7-10
Synthesis of results of individual studies	13	For all outcomes considered (benefits or harms), present, for each study: (a) summary of results and (b) relationship to other studies under review (e.g. agreements or disagreements in methods, sampling, data collection or findings).	10-16
**DISCUSSION**			
Summary of evidence	14	Summarize the main findings including the strength of evidence for each main outcome; consider their relevance to key groups (e.g., healthcare providers, users, and policy makers).	17
Limitations	15	Discuss limitations at study and outcome level (e.g., risk of bias), and at review-level (e.g., incomplete retrieval of identified research, reporting bias).	23
**CONCLUSION**			
Conclusions	16	Provide a general interpretation of the results in the context of other evidence, and implications for future research.	24

Free Sample downloaded from: https://guelphhumber.libguides.com/c.php?g=213266&p=1406923

Adapted from: Moher D, Liberati A, Tetzlaff J, Altman DG, The PRISMA Group (2009). Preferred Reporting Items for Systematic Reviews and Meta-Analyses: The PRISMA statement. *PLoS Medicine*, 6(6), e1000097. doi:10.1371/journal.pmed1000097

Supplementary material File S3. Search strategy

Pubmed

((Underrepresented [tiab] OR Underserved [tiab] OR Minority*[tiab] OR Diversity[tiab] OR Ethnic* [tiab] OR “Low socioeconomic”[tiab] OR Disadvantage* [tiab] OR Poor* [tiab] OR “Asian American”[tiab] OR “American Indian”[tiab] OR “Alaska Native”[tiab] OR “Native Hawaiian”[tiab] OR “Pacific Islander”[tiab] OR Hispanic[tiab] OR Latino[tiab] OR “African American”[tiab] OR Black[tiab] OR “Adolescent”[tiab] OR Teenager*[tiab] OR “Elderly people” [tiab] OR Woman*[tiab]) AND (Recruitment [tiab] OR (trial[tiab] AND (Participation [tiab] OR

Retention [tiab])) OR (recruitment[tiab] AND (barriers[TIAB] OR strategies[TIAB] OR opportunities[TIAB]))) AND ("Chronic Pain"[Mesh Terms] OR "experimental pain"[TIAB] OR "Chronic pain"[TIAB] OR "pain"[MESH Terms] OR "Low Back Pain"[Mesh] OR "Fibromyalgia"[Mesh] OR "Osteoarthritis"[Mesh] OR "Musculoskeletal Pain"[Mesh] OR "Neuralgia"[Mesh] OR "Low Back Pain"[tiab] OR "Fibromyalgia"[tiab] OR "Osteoarthritis"[tiab] OR "Musculoskeletal Pain"[tiab] OR "Neuralgia"[tiab] OR "neuropathic pain"[tiab] OR pain[tiab]))

Embase

('Underrepresented':ab OR 'Underserved':ab OR 'Minority':ab OR 'Diversity':ab OR 'Ethnic':ab OR 'Low socioeconomic':ab OR 'Disadvantage':ab OR 'Poor':ab OR 'Asian American':ab OR 'American Indian':ab OR 'Alaska Native':ab OR 'Native Hawaiian':ab OR 'Pacific Islander':ab OR 'Hispanic':ab OR 'Latino':ab OR 'African American':ab OR 'Black':ab OR 'Adolescent':ab OR 'Teenager':ab OR 'Elderly people':ab OR 'Woman':ab) AND ('Recruitment':ab OR ('trial':ab AND ('Participation':ab OR 'Retention':ab)) OR ('recruitment':ab AND ('barriers':ab OR 'strategies':ab OR 'opportunities':ab))) AND ('Chronic Pain'/exp OR 'experimental pain':ab OR 'Chronic pain':ab OR 'pain'/exp OR 'Low Back Pain'/exp OR 'Fibromyalgia'/exp OR 'Osteoarthritis'/exp OR 'Musculoskeletal Pain'/exp OR 'Neuralgia'/exp OR 'Low Back Pain'/exp OR 'Fibromyalgia':ab OR 'Osteoarthritis':ab OR 'Musculoskeletal Pain':ab OR 'Neuralgia':ab OR 'neuropathic pain':ab OR 'pain':ab)

Central

((Underrepresented:ti,ab OR Underserved:ti,ab OR Minority*:ti,ab OR Diversity:ti,ab OR Ethnic*:ti,ab OR "Low socioeconomic":ti,ab OR Disadvantage*:ti,ab OR Poor*:ti,ab OR "Asian American":ti,ab OR "American Indian":ti,ab OR "Alaska Native":ti,ab OR "Native Hawaiian":ti,ab OR "Pacific Islander":ti,ab OR Hispanic:ti,ab OR Latino:ti,ab OR "African American":ti,ab OR Black:ti,ab OR Adolescent:ti,ab OR Teenager*:ti,ab OR "Elderly people":ti,ab OR Woman*:ti,ab) AND (Recruitment:ti,ab OR (trial:ti,ab AND (Participation:ti,ab OR Retention:ti,ab)) OR (recruitment:ti,ab AND (barriers:ti,ab OR strategies:ti,ab OR opportunities:ti,ab))) AND ([mh "Chronic Pain"] OR "experimental pain":ti,ab OR "Chronic pain":ti,ab OR [mh pain] OR [mh "Low Back Pain"] OR [mh Fibromyalgia] OR [mh Osteoarthritis] OR [mh "Musculoskeletal Pain"] OR [mh Neuralgia] OR "Low Back Pain":ti,ab OR Fibromyalgia:ti,ab OR Osteoarthritis:ti,ab OR "Musculoskeletal Pain":ti,ab OR Neuralgia:ti,ab OR "neuropathic pain":ti,ab OR pain:ti,ab))

Table S1. List of excluded studies with reasons

Study	Title	Reason
Klem (2020)	What influences patient satisfaction after TKA? A qualitative investigation	Not recruitment
Amorim (2021)	Text Messaging and Web-Based Survey System to Recruit Patients With Low Back Pain and Collect Outcomes in the Emergency Department: Observational Study	Not underrepresented
Bastian (2020)	Stakeholder Engagement in Pragmatic Clinical Trials: Emphasizing Relationships to Improve Pain Management Delivery and Outcomes	Not recruitment
Minen (2020)	Smartphone based behavioral therapy for pain in multiple sclerosis (MS) patients: A feasibility acceptability randomized controlled study for the treatment of comorbid migraine and ms pain	Not underrepresented
Wylde (2020)	Screening to identify postoperative pain and cross-sectional associations between factors identified in this process with pain and function, three months after total knee replacement	Not recruitment
Eng (2017)	Preliminary evaluation of a new generation soft silicone foam dressing for the treatment of highly exudative venous leg ulcers (VLUS) under compression bandage	Not in the field of pain
Nikles (2021)	Pregabalin versus placebo to prevent chronic pain after whiplash injury in at-risk individuals: results of a feasibility study for a large randomised controlled trial	Not underrepresented
Bakshi (2021)	A pilot study of the acceptability, feasibility and safety of yoga for chronic pain in sickle cell disease	Not underrepresented
Miller (2020)	Patient and physician perceptions of knee and hip osteoarthritis care: A qualitative study	Not underrepresented
Wang (2021)	Participatory health through behavioural engagement and disruptive digital technology for postoperative rehabilitation: protocol of the PATHway trial	Not underrepresented
Sweeney (2021)	Pain management in inflammatory bowel disease: feasibility of an online therapist-supported CBT-based self-management intervention	Not underrepresented
Ohrtman (2019)	Longitudinal Clinical Trial Recruitment and Retention Challenges in the Burn Population: Lessons Learned from a Trial Examining a Novel Intervention for Chronic Neuropathic Symptoms	Not underrepresented
Whitaker (2021)	Laparoscopic treatment of isolated superficial peritoneal endometriosis for managing chronic pelvic pain in women: study protocol for a randomised controlled feasibility trial (ESPriT1)	Not a primary study

Jones (2021)	Interventions for promoting physical activity in people with neuromuscular disease	Not a primary study
Abuabara (2021)	International observational atopic dermatitis cohort to follow natural history and treatment course: TARGET-DERM AD study design and rationale	Not in the field of pain
Makris (2021)	Improving outcomes in older veterans with chronic low back pain and comorbid depression: Preliminary data from the motivate pilot trial	Not underrepresented
McKenna (2020)	The Impact of Exercise on Sleep in People with Rheumatoid Arthritis: A Pilot Randomised Controlled Trial	Not underrepresented
Glover (2020)	Healthy ageing in a deprived northern UK city: A co-creation study	Not in the field of pain
Jimenez (2021)	Feasibility and Acceptability of a Telephone-Based Intervention for Hispanic Children to Promote Treatment Adherence after Traumatic Brain Injury: A Pilot Study	Not in the field of pain
Kiarashi (2021)	Factors Associated With, and Mitigation Strategies for, Healthcare Disparities Faced by Patients With Headache Disorders	Not a primary study
Charleston (2020)	Equity of African American Men in Headache in the United States: A Perspective From African American Headache Medicine Specialists (Part 2)	Not a primary study
Nyring (2020)	Early migration of stemless and stemmed humeral components after total shoulder arthroplasty for osteoarthritis-study protocol for a randomized controlled trial	Not in the field of pain
Hefter (2020)	Disease Progression of Idiopathic Cervical Dystonia in Spite of Improvement After Botulinum Toxin Therapy	Not underrepresented
Beaulieu (2020)	Diarrhea, Abdominal Pain, and Weight Loss in Crohn's Disease: A Patient's Perspective	Not underrepresented
Lee (2015)	Determinants of ankylosing spondylitis related fatigue and the risks of its persistence	Not recruitment
Bodendorfer (2020)	Comparison of joint space measurements between supine and weightbearing anteroposterior pelvis radiographs for patients undergoing hip arthroscopy	Not in the field of pain
Minen (2020)	Are people with migraine willing to engage in digitally based behavioral therapies: A look at recruitment statistics for a mobile health study	Not underrepresented
Ho (2021)	Adaptations to Acupuncture and Pain Counseling Implementation in a Multisite Pragmatic Randomized Clinical Trial	Not underrepresented

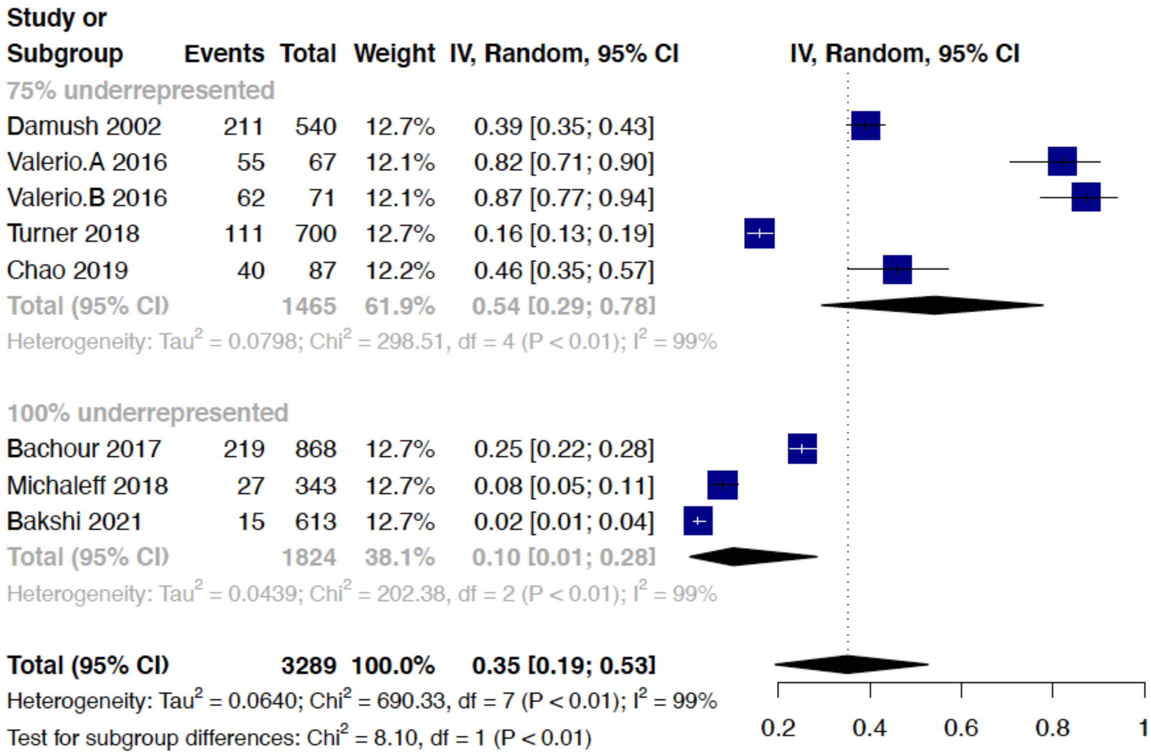

Figure S1. Sensitivity analysis of enrolment rate

Legend. Sensitivity analysis of enrolment rate divided between the studies including 100% and 75% of underrepresented populations on their sample [1-7].

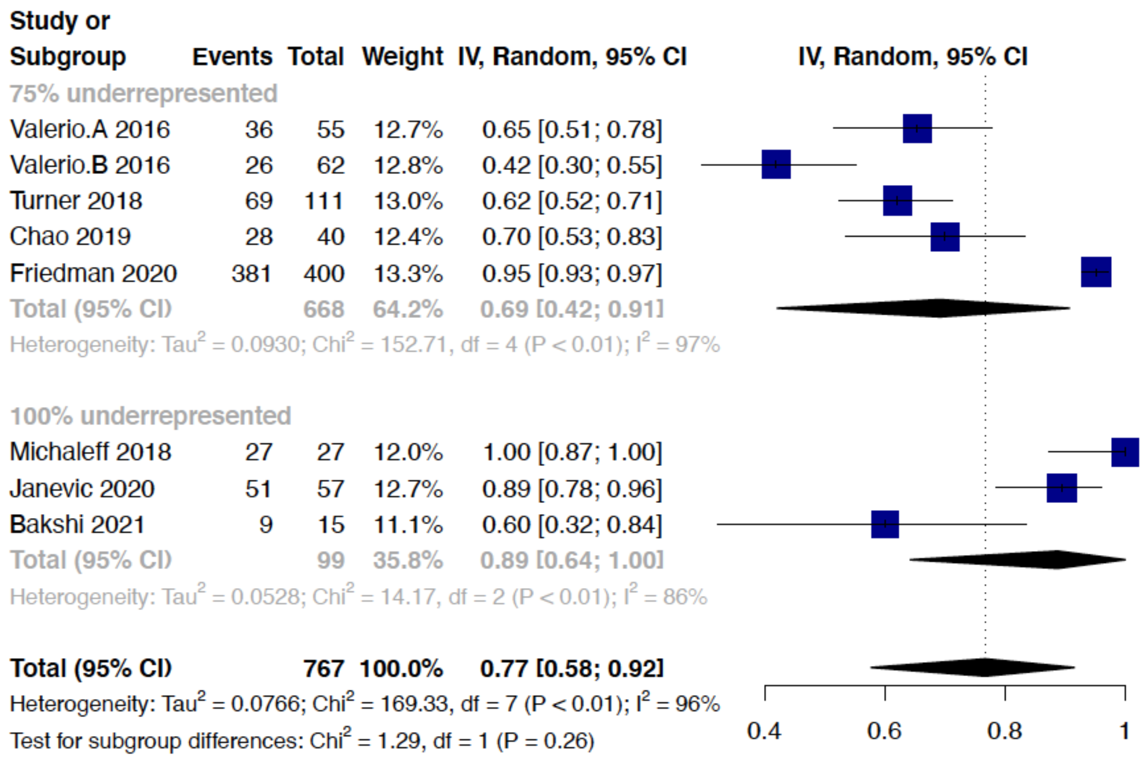

Figure S2. Sensitivity analysis of retention rate

Sensitivity analysis of retention rate divided between the studies including 100% and 75% of underrepresented populations on their sample [2-4,6-9].

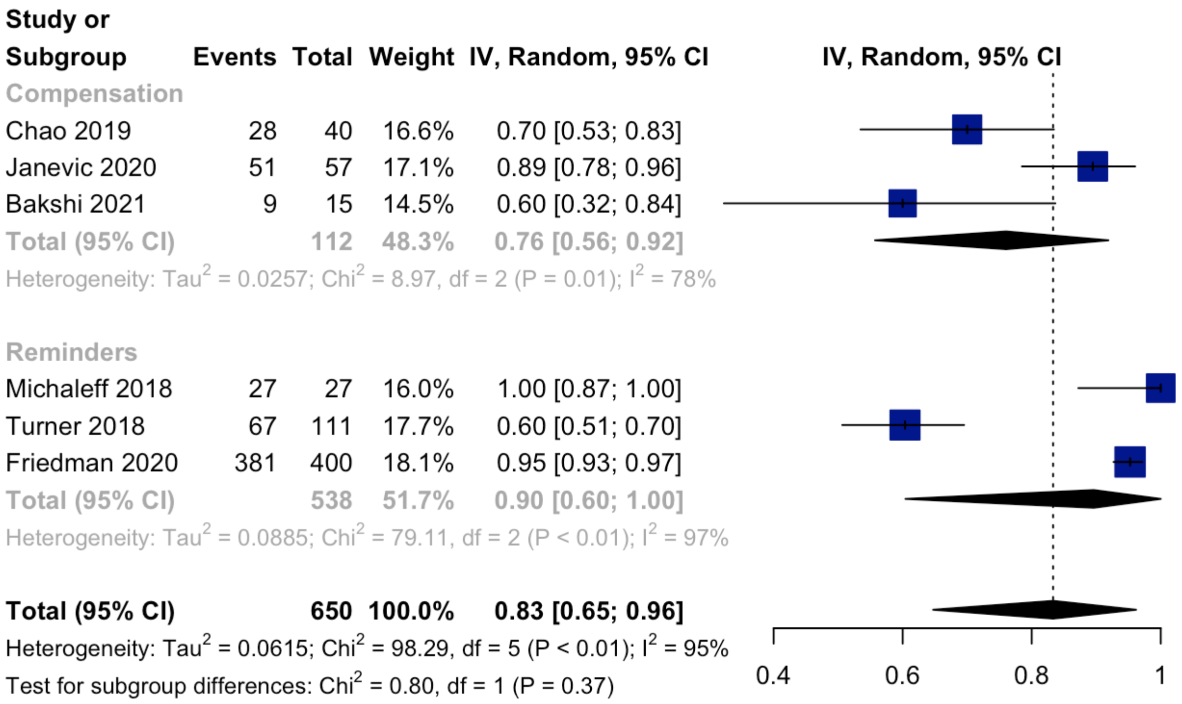

Figure S3. Forest plot of the retention strategies [3,4,6-9].

References

1. Damush, T.M.; Weinberger, M.; Clark, D.O.; Tierney, W.M.; Rao, J.K.; Perkins, S.M.; Verel, K. Acute low back pain self-management intervention for urban primary care patients: Rationale, design, and predictors of participation. *Arthritis Care & Research* **2002**, *47*, 372-379.
2. Valerio, M.A.; Rodriguez, N.; Winkler, P.; Lopez, J.; Dennison, M.; Liang, Y.; Turner, B.J. Comparing two sampling methods to engage hard-to-reach communities in research priority setting. *BMC Medical Research Methodology* **2016**, *16*, 146, doi:10.1186/s12874-016-0242-z.
3. Turner, B.J.; Liang, Y.; Simmonds, M.J.; Rodriguez, N.; Bobadilla, R.; Yin, Z. Randomized trial of chronic pain self-management program in the community or clinic for low-income primary care patients. *Journal of general internal medicine* **2018**, *33*, 668-677.
4. Chao, M.T.; Schillinger, D.; Nguyen, U.; Santana, T.; Liu, R.; Gregorich, S.; Hecht, F.M. A randomized clinical trial of group acupuncture for painful diabetic neuropathy among diverse safety net patients. *Pain Medicine* **2019**, *20*, 2292-2302.
5. Bachour, C.C.; Bachmann, G.A.; Foster, D.C.; Wan, J.Y.; Rawlinson, L.A.; Brown, C.S.; Gabapentin Study, G. Recruitment methods in a clinical trial of provoked vulvodynia: Predictors of enrollment. *Clinical Trials* **2017**, *14*, 103-108.
6. Michaleff, Z.A.; Campbell, P.; Hay, A.D.; Warburton, L.; Dunn, K.M. Child and adolescent musculoskeletal pain (CAM-Pain) feasibility study: testing a method of identifying, recruiting and collecting data from children and adolescents who consult about a musculoskeletal condition in UK general practice. *BMJ open* **2018**, *8*, e021116.

7. Bakshi, N.; Cooley, A.; Ross, D.; Hawkins, L.; Sullivan, M.; Astles, R.; Sinha, C.; Katoch, D.; Peddineni, M.; Gee, B.E. A pilot study of the acceptability, feasibility and safety of yoga for chronic pain in sickle cell disease. *Complementary therapies in medicine* **2021**, *59*, 102722.
8. Friedman, S.H.; Cunningham, C.O.; Lin, J.; Haramati, L.B.; Levsky, J.M. Having a primary care provider is the strongest predictor of successful follow-up of participants in a clinical trial. *The Journal of the American Board of Family Medicine* **2020**, *33*, 431-439.
9. Janevic, M.R.; Shute, V.; Murphy, S.L.; Piette, J.D. Acceptability and effects of commercially available activity trackers for chronic pain management among older African American adults. *Pain Medicine* **2020**, *21*, e68-e78.
